# Job burnout, cognitive functioning, and Brain-derived neurotrophic factor expression among hospital Mexican nurses

**DOI:** 10.1371/journal.pone.0304092

**Published:** 2024-05-24

**Authors:** Nadia Yanet Cortés-Álvarez, Alfredo Lara-Morales, Elizabeth Bautista-Rodríguez, Leticia Gabriela Marmolejo-Murillo, Alejandra Díaz Jiménez, Lourdes Alejandra Vergara Hernández, Monserrat Fernández Moya, César Rubén Vuelvas-Olmos

**Affiliations:** 1 Department of Nursing and Midwifery, Division of Natural and Exact Sciences, University of Guanajuato, Guanajuato, Mexico; 2 Faculty of Biotechnology, Laboratory of Medical & Pharmaceutical Biotechnology, Universidad Popular Autónoma del Estado de Puebla (UPAEP), Puebla, Mexico; 3 Department of Medicine and Nutrition, Division of Health Sciences, University of Guanajuato, Guanajuato, Mexico; 4 Psychology Program, University Rosario Castellanos, Mexico City, Mexico; 5 Department of Nursing and Midwifery, Division of Life Sciences, University of Guanajuato, Guanajuato, Mexico; 6 Medical Sciences Program, School of Medicine, University of Colima, Colima, Mexico; University of Huelva: Universidad de Huelva, SPAIN

## Abstract

**Aim:**

To analyze the relationship between burnout syndrome, cognitive functions, and sBDNF (Serum Brain-derived Neurotrophic Factor) in Mexican nurses.

**Method:**

A descriptive cross-sectional design was used. This study target staff nurses working in hospitals in Guanajuato, México. Demographic and working condition data were collected via questionnaire. The Maslach Burnout Inventory (MBI) was used to evaluate burnout. A blood sample were collected and processed by ELISA technique to measure sBDNF. Finally, the General Cognitive Assessment (CAB) of the Cognifit© neuropsychological battery was used to evaluated cognitive functions.

**Results:**

Findings showed that there are sociodemographic characteristics and working conditions associated with burnout syndrome among nurses. Furthermore, the data demonstrated a significant decrease in sBDNF levels in burnout nurses and a negative correlation between BDNF levels and burnout syndrome. Additionally, these burnout nurse also revealed significant cognitive impairment in reasoning, memory, and attention as well as total scores of CAB. Interestingly, we found a positive correlation between sBDNF levels and the cognitive deficits in burnout nurse.

**Conclusion:**

Reduced BDNF levels could be a biological indicator or part of the pathological process of burnout, which could affect cognitive abilities. Reduced cognitive function in nurses has relevant implications and emphasizes the need for specialized preventive strategies because nurses make clinical decisions concerning their patients, whose situations are constantly changing.

## 1. Introduction

The nursing profession has historically been stressful due to a variety of factors, including physical labor, patient suffering, death, staffing issues, disagreements with coworkers, insufficient professional training, a lack of social support, an overwhelming workload and demands at work, the growing use of technology, the unpredictability of patient care and the unsettling mismatch between expectations and the reality of nursing as a profession [1,2]. Additionally, the health crisis caused by the COVID-19 pandemic, where nurses, as the largest collective of health professionals, worked under enormous pressure, with limited protective supplies [3,4] and high work demands, which together have represented a risk for triggering or aggravating the presence of burnout syndrome.

Burnout syndrome has conceptualized as a syndrome in response to chronic (long-term and cumulative) stress at work. The main symptoms of burnout syndrome include emotional exhaustion, depersonalization, and reduced personal achievement [5]. Health personnel suffering from burnout syndrome may present negative personal consequences such as substance abuse, drug dependence, strained relationships, depression, anxiety, and even suicidal ideas [6,7]. Furthermore, affecting important professional consequences such as lower patient satisfaction, deteriorated quality of care, decreased work productivity, and an increased risk of laxity [8–11], resulting in nurses being susceptible to negligence lawsuits with substantial costs for health personnel and hospitals [12].

Alarmingly, one of the main symptoms of burnout syndrome is cognitive impairment [13–15], with predominant deficits related to executive functions, attention, and memory [16,17], which may predispose nurses to medical errors [18] due to practicing nursing involves complex cognitive processes such a clinical reasoning, making inferences and decision-making to determine an appropriate course of action [19]. These cognitive functions are related to the coordination and regulation of thoughts, behavior towards chosen goals, and the ability to switch between multiple tasks [20,21], as well as visuospatial construction skills [22].

Brain-derived neurotrophic factor (BDNF) is a neurotrophin family protein that is widely expressed in the hippocampus, hypothalamus, and cerebral cortex. It is involved in the survival, growth, and maintenance of several types of neurons as well as synaptic plasticity [23]. Furthermore, neurogenesis and long-term potentiation—two processes suggested to underlie biological mechanisms for hippocampal-dependent forms of learning and memory [24,25]—have been linked to decreased levels of BDNF [26–28].

Research has suggested that reduced BDNF levels may be linked to the neurobiology of burnout syndrome and are linked to cognitive deficits [29]. Research has shown that individuals with burnout syndrome tend to have low levels of sBDNF and that depersonalization and emotional weariness are negatively correlated. Yet, there is also an indication of a positive correlation with the competence subscales of the burnout inventory, suggesting that low BDNF may contribute to the neurobiology of burnout syndrome [13,29].

Given the importance of the nursing staff in all health care systems and considering that it is one of the most exposed sectors to psychosocial illnesses, the aim of the present study was to analyze the relationship between burnout syndrome, cognitive functions, and sBDNF in nursing personnel.

## 2. Methods

### Design

A descriptive cross-sectional design was used.

### Participants and settings

This study target staff nurses working in hospitals in Guanajuato, México. A convenience sampling technique was used to select study hospitals. Both sexes, at least a nursing diploma, a minimum of one year in the present unit, and signing the informed consent form were requirements for inclusion. Participants were not required to have directly cared for patients with COVID-19. Nurses who did not adequately or thoroughly fulfil the survey form were excluded. All completed the survey in February 2022.

### Data collection

A research invitation to take part in the study was put up on the hospital bulletin board once the managers of the hospitals gave their approval. An online questionnaire in Spanish was designed and applied through an online survey platform (Google Forms, Google Inc., California, USA). Each head nurse received the survey link, which they then shared with the hospital’s nursing WhatsApp group. Nurses were invited to spread the word about the study to as many nurses as possible. The survey assessed three types of variables: demographic characteristics, working condition and burnout syndrome.

○ **Demographic variables**: Professional license number and hospital workplace (to make sure they met the inclusion criteria and avoid duplication of respondents), age, sex, and marital status.
○ **Working condition**: level education, years of experience in the profession, nurse‐to‐patient ratio, if consider appropriate the number of patients assigned per day, type of hiring, hours worked a week, work shift, number of current Jobs (by the time of the study), dependents, and job satisfaction.
○ **Burnout syndrome**: The Maslach Burnout Inventory (MBI), of Maslach and Jackson was used, MBI is a 15-item self-report survey with a 7-point rating system (0 = never to 6 = every day), which assess the three burnout dimensions (emotional exhaustion (EA), depersonalization (DP), and reduced professional fulfillment (RP)) [30]. Each dimension’s total scores are categorized into high, moderate, and low tertiles. The dimension measuring EA has three cutoff points: low <11, moderate 11–15, and high ≥ 15. There are three strata for the DP subscale: low <9, moderate 9–12, and high ≥ 12. Lastly, the cut-off points for the RP dimension are low <19, moderate 19–22, and high ≥ 22 [13]. Scores high in AE and DP and low in RP define the syndrome [31,32]. The MBI Inventory has been translated into Spanish and has shown satisfactory reliability and validity; the internal consistency coefficient of the three dimensions was 0.835, 0.607, and 0.733.

### Measurement of sBDNF levels

Following an overnight fast, participants’ blood samples were taken. Serum was separated and processed by sandwich enzyme-linked immunosorbent assay (ELISA technique) using a commercial kit (Human BDNF ELISA Kit ab212166 | Abcam) according to the manufacturer’s instructions.

### Cognitive evaluation

The General Cognitive Assessment (CAB) of the Cognifit© neuropsychological battery was administered the same day the blood sample was drawn (https://www.cognifit.com/es). All subjects performed the test at least one hour after breakfast. Cognifit© is used individually to measure the cognitive functioning of all participants. The cognitive functions evaluated were reasoning, memory, attention, coordination, and perception. Each of these cognitive functions had subtasks, which were also evaluated.

### Ethical considerations

This study was conducted in compliance with the Norma Oficial Mexicana-012-SSA3-2012 and Declaration of Helsinki. Additionally, it was approved by the Research and Bioethics Committee of the Hospital Materno Infantil de Irapuato (Registration number CIBHMII-004). All participants gave their informed consent. During the collection and analysis of the data, the principles of confidentiality and privacy were ensured while maintaining the anonymity of the participants.

### Data analysis

Sample characteristics were described using basic statistics, including means, standard deviations, frequencies, and percentages. Multivariate regression analysis with the ’enter’ method to delve into how BDNF levels and Cognitive Functions performance in Cognifit influenced in Burnout (MBI Burnout). Univariate generalized linear with a main effects model (linear regression) was used to measure the associations between sociodemographic characteristics and working conditions with MBI scores. Associations were presented using beta coefficients, confidence intervals, and P-values. The significance level was set at 0.05. Analyses were performed in IBM SPSS Statistics (version 26.0).

## 3. Results

### Burnout syndrome, sociodemographic characteristics and working condition

According to the results obtained among the Maslach Burnout Inventory, participants were divided in two groups: With burnout syndrome (n = 50) and without syndrome (n = 50). Table 1 shows the sociodemographic characteristics and working conditions among both groups.

**Table 1:** Sociodemographic characteristics and Working conditions.

Variables	With syndrome burnout	Without syndrome burnout
**Sex** ^a^		
Women	25 (50)	25 (50)
Men	25 (50)	25 (50)
**Marital status** ^a^		
Married	22 (44)	23 (46)
Divorced	7 (14)	5 (10)
Single	12 (24)	19 (38)
Common-law marriage	6 (12)	2 (4)
Widowed	3 (6)	1 (2)
**Age (years)** ^b^	37.92 ± 7.34	37.30 ± 6.16
**Highest educational level** ^a^		
Technique	13 (26)	14 (28)
College	28 (56)	30 (60)
Especiality	7 (14)	5 (10)
Posgrade	2 (4)	1 (2)
**Experience in the profession (years)** ^a^		
≤10 years	15 (30)	13 (26)
≥11 years	35 (70)	37 (74)
**Nurse‐to‐patient ratio** ^a^		
1:1	1 (2)	3 (6)
1:2	1 (2)	3 (6)
1:3	3 (6)	4 (8)
1:4	3 (6)	5 (10)
1:5	19 (38)	16 (32)
1:6 or more	23 (46)	19 (38)
**Considers appropriate the number of patients assigned per day** ^a^		
Yes	5 (10)	10 (20)
No	45 (90)	40 (80)
**Type of hiring** ^a^		
Temporary contract	15 (35)	20 (40)
Indefinite contract	35 (70)	30 (60)
**Hours worked a week** ^a^		
Up to 24 hours	2 (4)	6 (12)
Up to 36 hours	4 (8)	9 (18)
Up to 40 hours	17 (34)	14 (28)
More than 48 hours	27 (54)	21 (42)
**Work shift** ^a^		
Morning	2 (4)	5 (10)
Evening	4 (8)	6 (12)
Night	9 (18)	8 (16)
More than one work shift*	23 (46)	21 (42)
Rotating**	12 (24)	10 (20)
**Number of current jobs (by the time of the study)** ^a^		
1 job only	8 (16)	11 (22)
2 jobs	22 (44)	20 (40)
3 jobs	15 (30)	13 (26)
More than 3 jobs	5 (10)	6 (12)
**Dependents** ^a^		
0 to 3 people	33 (66)	37 (74)
4 to 6 people	13 (26)	10 (20)
More than 7 people	4 (8)	3 (6)
**Job satisfaction** ^a^		
Dissatisfied	17 (34)	12 (24)
Little satisfied	23 (46)	27 (54)
Satisfied	6 (12)	5 (10)
Very Satisfied	4 (8)	6 (12)

^a^Data presented as frequencies and percentages.

^b^Data presented as mean ± standard deviation.

^c^Comparison using the X^2^ test.

^d^Comparison using the t-test.

*Those people who work more than one work shift per day.

**Those people who just have one work shift, but it is not a fixed shift.

### Association between sociodemographic characteristics and working condition on burnout syndrome

As shown Table 2, female nurses, married individuals, those with a college education or higher, and nurses with over 11 years of experience are more likely to report higher Maslach Burnout Inventory (MBI) scores, indicating an increased risk of burnout syndrome. Other factors significantly associated with elevated MBI scores include higher nurse-to-patient ratios, temporary contracts, longer working hours (more than 48 hours per week), and certain work shift patterns (rotating or multiple shifts).

**Table 2:** Associations between demographic data, depression, anxiety, sleep quality, physical activity, and cognitive function (second evaluation).

Variable	MBI Score		
	β	95% CI	p-value
**Sex**			
Female	3.852	0.620 to 5.084	**0.008** *
Male	Reference		
**Marital status**			
Married	2.752	1.421 to 3.083	**0.023** *
Divorced	0.169	-0.315 to 0.653	0.497
Single	1.203	1.192 to 2.514	**0.001** *
Common-law marriage	1.034.	-0.421 to 1.353	0.931
Widowed	Reference		
**Highest educational level**			
Technique	0.875	0.632 to 1.118	0.051
College	1.234	1.014 to 2.454	**0.007** *
Especiality	0.423	0.198 to 0.648	0.633
Posgrade	Reference		
**Experience in the profession (years)**			
≥11 years	1.315	1.045 to 1.586	**0.002** *
≤10 years	Reference		
**Nurse‐to‐patient ratio**			
1:6 or more	3.215	1.015 to 4.415	**0.004** *
1:5	2.452	1.214 to 3.690	**0.011** *
1:4	0.768	0.591 to 0.946	0.071
1:3	0.874	0.732 to 7.016	0.426
1:2	0.237	0.364 to 0.8237	0.837
1:1	Reference		
**Type of hiring**			
Temporary contract	1.327	1.035 to 1.619	**0.008** *
Indefinite contract	Reference		
**Hours worked a week**			
More than 48 hours	4.576	1.289 to 6.863	**0.001** *
Up to 40 hours	1.423	1.128 to 1.718	0.204
Up to 36 hours	0.774	0.374 to 0.947	0.375
Up to 24 hours	Reference		
**Work shift**			
Rotating	2.734	1.352 to 3.116	**0.001** *
More than one work shift	1.412	1.124 to 2.701	**0.006** *
Night	1.237	0.975 to 1.499	**0.032** *
Evening	0.869	0.674 to 1.065	0.141
Morning	0.935	0.726 to 1.144	0.208
**Number of current jobs (by the time of the study)**			
More than 3 jobs	2.876	1.463 to 4.290	**0.001** *
3 jobs	1.523	1.210 to 2.836	**0.003** *
2 jobs	0.847	0.625 to 1.070	0.158
1 job only	Reference		
**Dependents**			
More than 7 people	2.327	1.014 to 3.640	**0.007** *
4 to 6 people	1.542	1.208 to 2.876	**0.001** *
0 to 3 people	0.865	0.632 to 1.098	0.106
Dependents	Reference		
**Job satisfaction**			
Dissatisfied	3.876	1.463 to 4.290	**0.001** *
Little satisfied	2.523	1.210 to 3.836	**0.003** *
Satisfied	0.847	0.625 to 1.070	0.327
Very Satisfied	Reference		

β: Beta coefficient; CI: Confidence Interval; *p<0.05

**p<0.001.

### Cognitive functions

**Fig 1:**
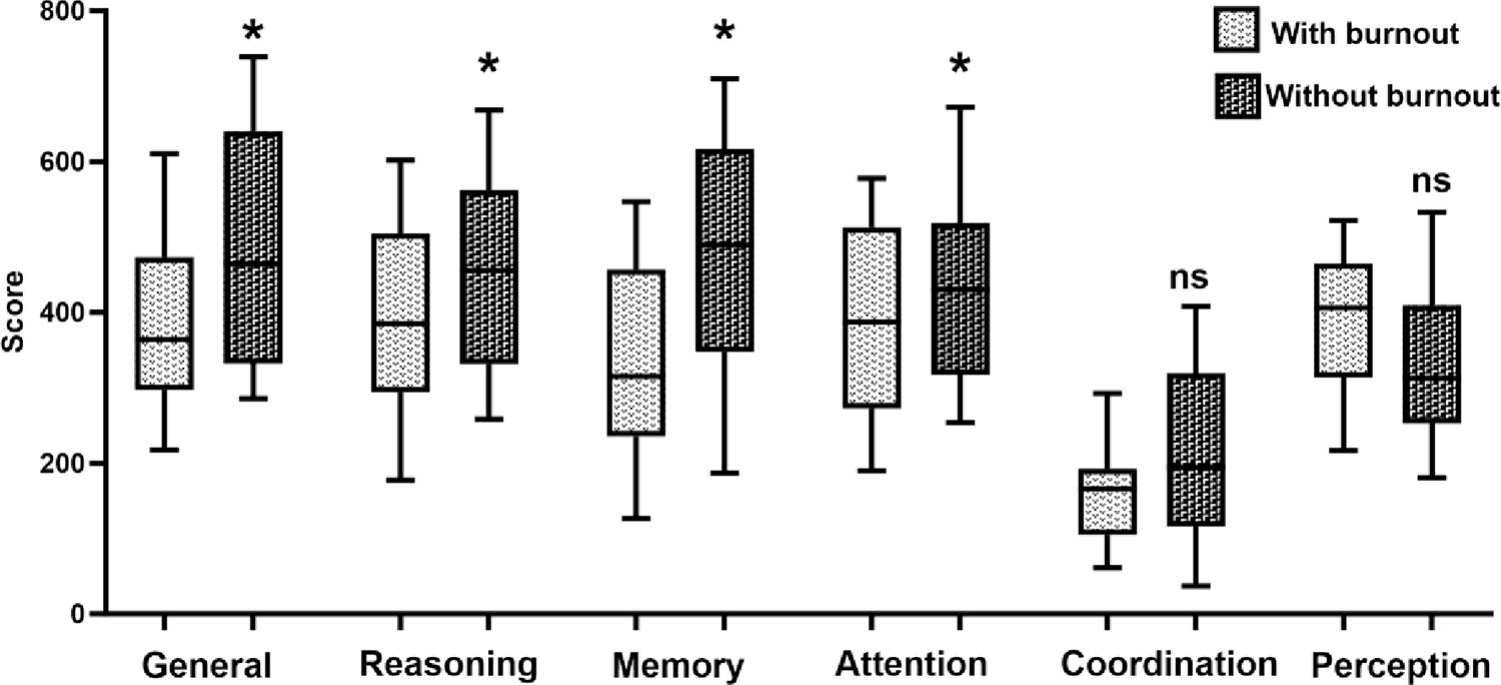
Comparing cognitive outcomes: Performance in task Cognifit. White box with a point = nurses with burnout. Black box with point = nurses without burnout. Unpaired t-tests were used to measure statistical significance *p<0.05.

**Fig 2:**
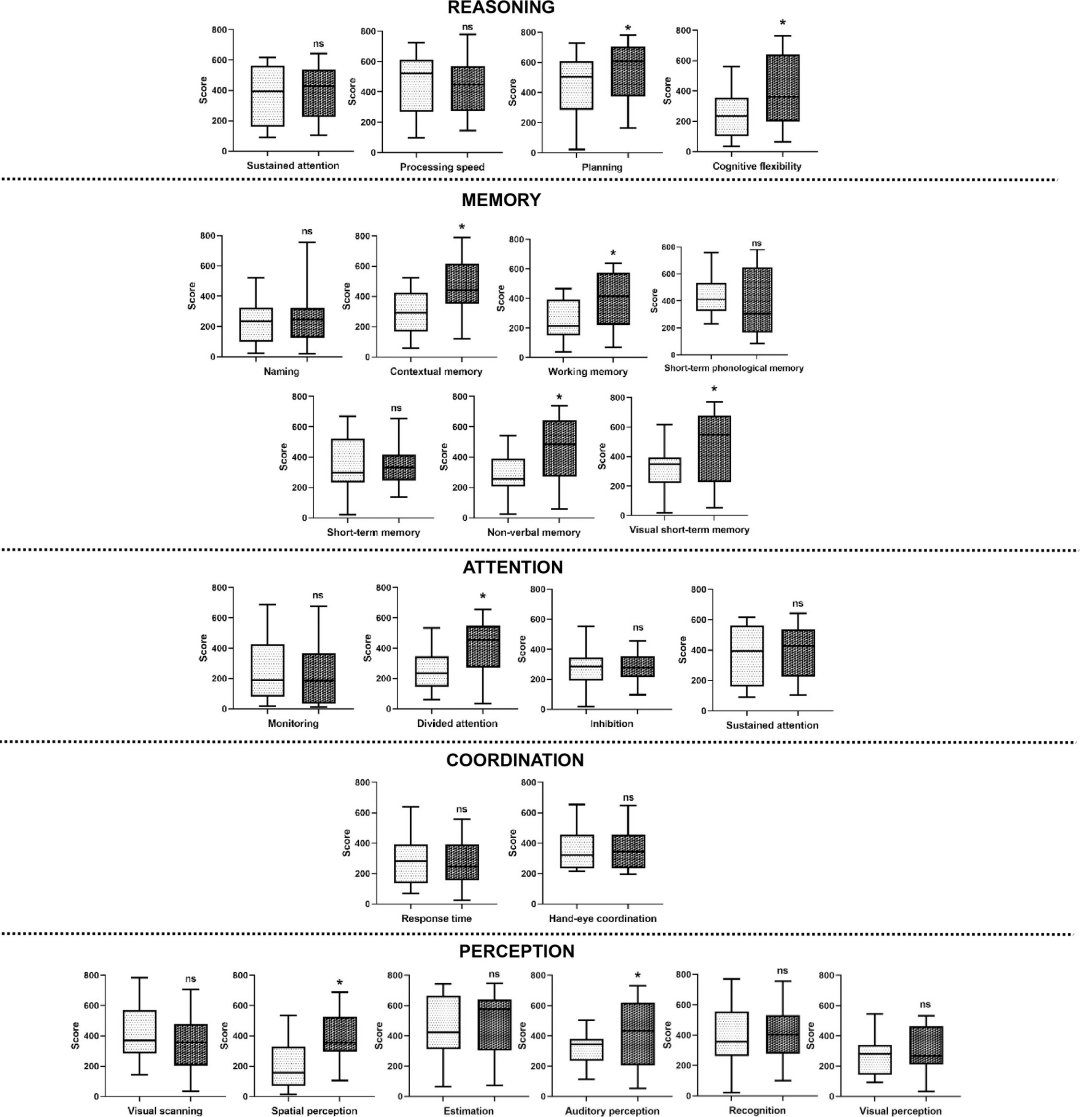
Comparing sub cognitive task: Performance in Cognifit. White box with a point = nurses with burnout. Black box with point = nurses without burnout. Unpaired t-tests were used to measure statistical significance *p<0.05.

As shown in Fig 1, when comparing both groups, a statistically significant difference was identified in total scores of CAB, reasoning, memory, and attention, revealing lower scores in the group of nurses without burnout syndrome. Similarly, Fig 2 shows lower scores in the planning, cognitive flexibility, contextual memory, working memory, nonverbal memory, visual short-term memory, divided attention, spatial perception, and auditory perception subtasks on group of nurses without burnout syndrome.

### sBDNF levels

**Fig 3:**
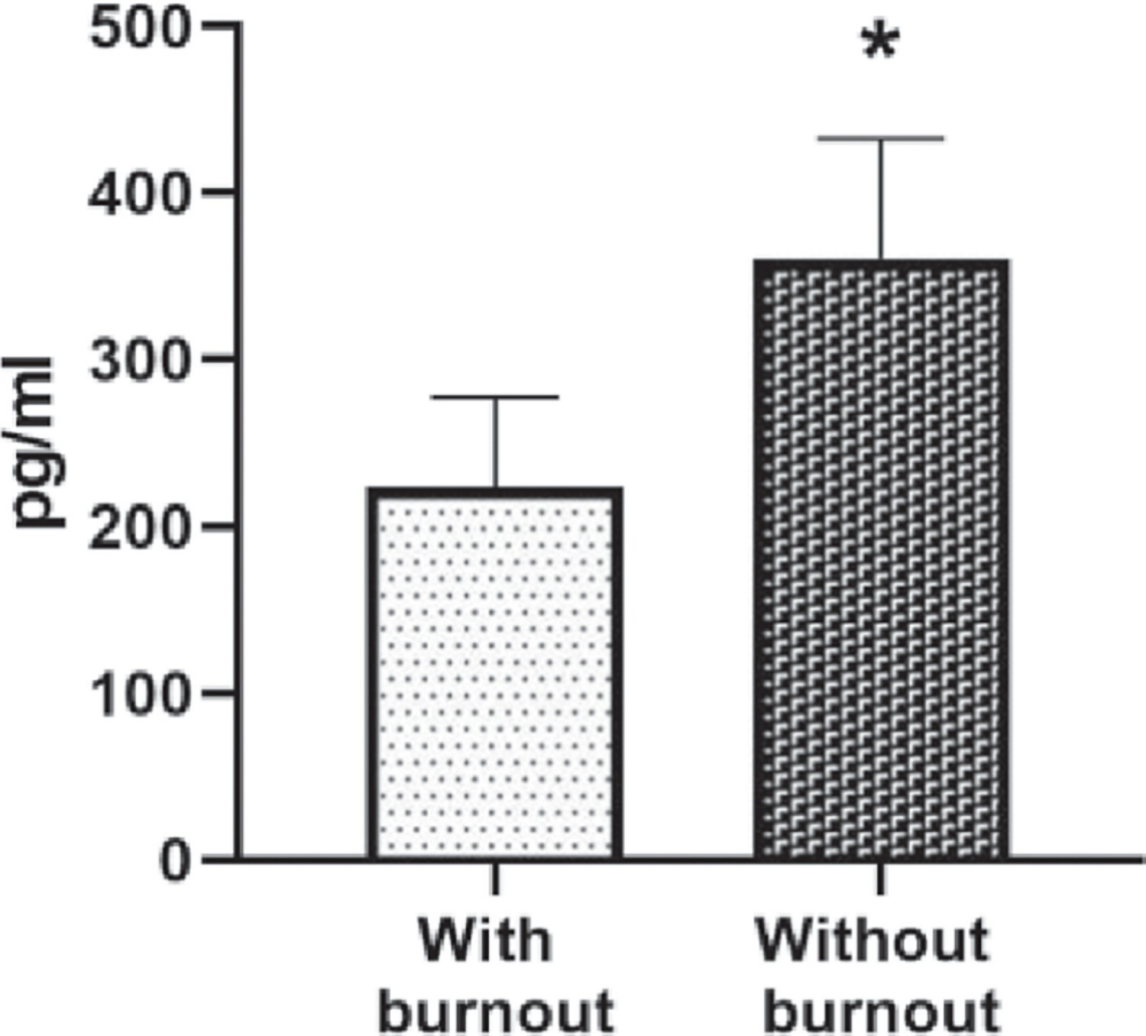
sBDNF levels among both groups nurse staff. Unpaired t-tests were used to measure statistical significance *p<0.05.

As shown in Fig 3, a significantly lower expression was identified (t = 3.248, df = 46, p = 0.001) in the nursing group with burnout syndrome (243.17 ±84.49 pg/ml) compared to group without burnout syndrome (362.75 ±68.79 pg/ml). Furthermore, a negative correlation was identified between BDNF levels and burnout syndrome (r = −.414; p<0.001).

### Linear regression analysis of burnout syndrome and its relationship with BDNF and cognitive functions

The multiple regression analysis results indicate that BDNF levels and cognitive functions (reasoning and memory) are significantly negatively associated with the MBI score. This means that higher BDNF levels and cognitive functions are associated with lower MBI scores. The independent predictors attention, coordination, and perception were not significantly associated with the MBI score (Table 3).

**Table 3:** Linear regression model with MBI score.

	MBI Score		
	*B*	95% CI	p-value
**BDNF**			
pg/ml	1.432	0.620 to 0.935	**0.001** *
**Cognitive functions**			
Total scores of CAB	0.871	0.364 to 0.953	**0.006** *
Reasoning	0.832	0.421 to 0.926	**0.015** *
Memory	0.671	0.238 to 0.833	**0.027** *
Attention	0.345	0.192 to 0.871	0.359
Coordination	0.523	0.421 to 0.715	0.931
Perception	0.148	0.094 to 0.423	0.822

β: Beta coefficient; 95% CI: Confidence Interval; *p<0.05.

### Correlation between BDNF levels and cognitive functions among burnout group

Finally, we analyzed the correlation coefficients (r) and p-values for the correlation between BDNF levels and cognitive function. Findings shown that there is a significant positive correlation between BDNF levels on general cognitive function, as well as memory, attention, and reasoning (Table 4).

**Table 4:** Correlation between cognitive function data and BDNF protein expression among burnout group.

Cognitive evaluation	BDNF Levels	
	r	*p*
Total scores of CAB	0.591	0.007*
Reasoning	0.564	0.003*
Memory	0.541	0.005*
Attention	0.615	0.001*
Coordination	0.102	0.482
Perception	0.121	0.615

Data analyzed using Pearson’s correlation coefficient. N = 50.

## 4. Discussion

The origin of research on burnout syndrome occurs in professions involving human interactions, particularly those that require attending to individuals in need of assistance and care, including nursing personnel. Based on this background, the aim of the present study was to analyze the relationship between burnout syndrome, cognitive functions, and sBDNF among nurses with and without burnout.

In the present study burnout syndrome were associated with female sex, more than 4 dependents, as well as have married and single marital status These findings are consistent with a previous literature since it was found how females suffered higher levels of burnout syndrome than males in their working environment [33,34]. Furthermore, consistent with other research [35,36], burnout was more common among female nurses than male nurses. In general, women have more combined duties at work and home than men do, which may be the source of this condition [37]. Additionally, it has been proposed that after repeated stressors, women are still more susceptible than males to lower cortisol levels, women may therefore be more sensitive than men to mood dysregulation after psychosocial stress due to the changes in ovarian hormones on the hypothalamic-pituitary-adrenal (HPA) axis and brain circuits involved in the stress response [38].

Regarding the number of dependents, it has been suggested that a higher number of children or economic dependents has been associated with higher levels of burnout among health personnel [39,40]. Number of dependents, there has been a proposal indicating that an increased count of children or economic dependents is associated with elevated instances of burnout among healthcare personnel [41]. The observed outcomes could be elucidated by the dual-role responsibilities individuals undertake, managing both their professional commitments and domestic roles within society. Some studies show that even the presence of burnout is more intense in those professionals who have children under 12 years of age, attributable to an increased need for supervision and care at home [42]. The simultaneous assumption of several responsibilities can lead to a greater perception of individual burnout, especially when they have a greater number of children [43].

Finally, in the present study, both the married and the single marital status was associated with higher levels of burnout syndrome. These findings are replicated in other studies, for example Hameed et al. 2018 [44] suggest that married status increased the chance of burnout by 2.64 times compared to unmarried residents while Hompoth et al. 2018 [45] report that being single was a risk factor for burnout. These contradictory findings could be explained by the fact that, on the one hand marriage comes with additional responsibilities which could contribute to burnout but not having a partner can mean lack of support security, and encouragement that the couple offers to cope with work-related stressors.

On the other hand, college education, ≥11 years’ experience in the profession, have 3 or more jobs, 1:5 or more nurse-to-patient ratio, temporary contract, and work more than 48 hours a week were associated with higher burnout syndrome. Additionally, work on night shift, more than one work shift and rotating work shift, and little or no job satisfaction also were associated with higher burnout syndrome.

Although it has been reported that, nursing staff see the night shift as a good component of their life because they are able to balance their schedules and organize their personal and professional lives, it is a fact, however, that working the night shift throws off employees’ biological cycles, which can result in both physical and mental illnesses [46,47]. Research has shown that the circadian system is resistant to changing from a day-to-night schedule; this is demonstrated by the absence of significant phase shifts over several days in centrally controlled rhythms, such melatonin and cortisol [48].

In fact, Cannizzaro et al. 2020 [49] report night-time shift cortisol levels significantly increased before and after the work shifts. In the same line, Ibar et al., 2021 [50] found a direct relationship between hair cortisol concentration and both perceived stress and emotional exhaustion. Moreover, particularly in Mexico, despite its important and valuable work, nursing staff work under precarious conditions. According to the analysis of Aristizabal et al. 2019 [51], four of the five labor precariousness indicators among nurses in Mexico showed an increase: the percentage of people without a written contract, the percentage of people whose income is less than twice the minimum wage, the percentage of nurses without social security, and the percentage of nurses without social benefits. Also, authors revealed that, from 46% in 2005–2006 to 54% in 2018, the proportion of nurses who work in precarious conditions.

In the same line, the monthly salary that a Mexican nurse receives is one of the lowest in the world [52], which forces them to exceed the work limit (48 hours per week) established by the Federal Labor Law [53], whether in the same job or with several jobs simultaneously. Of note, adverse job characteristics could influence the job satisfaction, and a dissatisfaction job could be associated with burnout [54,55].

As suggested in previous studies, inadequate staffing is an important factor in the experience of nurse burnout. In this line, the patient-to-nurse ratio has been linked to burnout, indicating that the higher the patient-to-nurse ratio, the higher the burnout syndrome [56,57]. Alarmingly, one study concluded that burnout mediated the relationship between patient-to-nurse ratios and mortality and patient safety [58,59]. In fact, it has even been suggested that burnout can be reduced by decreasing the nurse-patient ratio, which would allow nurses to have the time and energy necessary to care for patients and their families [60]. The patterns identified previous [39,54,55,61], and the present study consistently show that complex work environments are considered an important factor in burnout development, and even this factor could be decisive for staff to decide to leave their jobs.

Also, our results showed significantly lower expression BDNF in the nursing group with burnout syndrome compared to the group without burnout syndrome, furthermore, a negative correlation between BDNF levels and burnout syndrome was identified. These finding supports previous studies that have suggested that chronic stress can lead to reduced BDNF levels. For example, subjects suffering from burnout syndrome had significantly lower BDNF levels [13,62]. Similarly, according to He et al. [63], there may be a connection between sBDNF levels and job stress, causing individuals with lower sBDNF levels to experience higher degrees of job burnout.

Among the hypothesized neurobiological pathways explaining how BDNF affects burnout, it has been suggested to be due to the impact of BDNF on hippocampal synaptic plasticity. More precisely, BDNF may control high-frequency synaptic transmission, which involves MAPK and PI3K. Nevertheless, it has been discovered that impairment of the structure and function of the hippocampus is linked to mental illnesses such as anxiety and depression [63,64]. Furthermore, it was found that BDNF, which has a close relationship to the dopaminergic system’s, activates the TrkB receptor, increasing dopamine release in the nucleus accumbens. It is widely acknowledged that a variety of mental diseases are significantly influenced by anomalies in the dopaminergic system [65].

At the genetic level, previous research has demonstrated that gene polymorphisms affect BDNF secretion, and individuals with distinct genotypes exhibit varying levels of BDNF [63]. He et al.’s study [66], for instance, showed that the BDNF rs2049046 altered the levels of sBDNF, indicating that the TT homozygote had a lower amount of sBDNF than the AA and AT genotypes. According to this, the authors hypothesize that a combination of BDNF rs2049046 and job stress led to a greater degree of job burnout in AT genotype patients compared to AA homozygote subjects [66]. Together, these findings lead us to hypothesize that reduced sBDNF could be a biological sign or potentially a pathological mechanism of burnout. However, more research is needed to ascertain the relationship between sBDNF levels and burnout.

Furthermore, when comparing the burnout group to its counterparts, our results revealed cognitive changes on total scores of CAB, reasoning, memory, and attention, without change in perception and coordination. The results of our current study, which showed worse cognitive performance in the burnout group, are consistent with most of the earlier research assessing cognitive function in the burnout [67–71]. For example, finding of Chutko et al. [68] indicate cognitive complaints in burnout syndrome patients, while research by Rudman et al. [69], showed that high levels of burnout symptoms were significantly linked to more frequent symptoms of cognitive dysfunction in nurses, both early in their careers and later in life. Similarly, Pihlaja et al. [70] reported that subjects with burnout had more challenges in executive functions, than those without it and finally, according to Gavelin et al. [71] clinical burnout may be linked to decreased functioning in executive function, working memory, episodic memory, attention, and processing speed. While most of the reviewed research demonstrates cognitive deficits, it is important to emphasize that there is disagreement amongst them about the specific cognitive areas that are vulnerable to the potential effects of burnout and the degree of cognitive impairments.

A plausible rationale is related to the pathogenic mechanisms involved in burnout. There is a notion that stress relates to cognitive deficits in burnout [72]. The brain is susceptible to stress, and aside from the hypothalamus, the hippocampal area was the first to be identified as a target of glucocorticoids (e.g., cortisol) [73].

Long-term stress adaptation is regulated by the HPA axis, which requires effective termination of the stress response through a negative feedback mechanism including the action of cortisol on limbic circuits such as the hippocampus, amygdala, and prefrontal cortex, and burnout impacts these neuronal networks, this lowers the efficiency of this process and, as a result, increases basal cortisol output [74]. These neural networks can be harmed by prolonged cortisol hypersecretion, sculpting dendrites and synapses [75]. Cognitive processing requires the integrity of areas such as the hippocampus, amygdala, and prefrontal cortex [73], and stress can affect its functioning by regulating cortisol levels [76]. Therefore, cognitive deficiencies in burnout may be caused by stress-induced damage to brain regions.

Likewise, as previously described, numerous studies have demonstrated that chronic stress can lead to reduced BDNF [13,62,63] and it is well known that BDNF is a neurotrophin considered as a biomarker of cognitive function [77–79] due to being essential for controlling synaptic plasticity and promoting dopaminergic neuron survival [80], also it is considered an important regulator of synaptic transmission and LTP in the hippocampus and in other brain regions and are involved in hippocampal neurogenesis [81].

Although other research has shown a positive correlation between sBDNF levels and cognitive function in both healthy people and certain disorders that impair cognition, [82,83], as far as we know, not many studies are currently available that have analyzed the relationship between BDNF and cognition on burnout subjects. On the literature search, only found the article of He et al. [13] who reported a positive correlation between sBDNF and the cognitive total score, immediate memory, and attention in burnout participants. The relationship between BDNF and cognition on burnout subjects is supported by the present study, which identified a positive correlation between sBDNF and total scores of CAB, reasoning, memory as well as attention. Taken together, the current research supports the theory put forth by He et al. [13], which contends that chronic stress mediated by HPA axis dysfunction in burnout may be linked to lowered BDNF levels. This, in turn, may lead to inadequate brain plasticity by lowering hippocampal neurogenesis through lowered BDNF levels, potentially resulting in cognitive impairments.

In conclusion, our research demonstrated that certain sociodemographic characteristics and workplace circumstances are linked to burnout syndrome in nurses. It is necessary that these factors be considered for implementation of important clinical and policy strategies which support the daily work of nurses. In addition, findings showed a significantly decreased of sBDNF levels in burnout nurse as well as negative correlation between BDNF levels and burnout syndrome. Taken together, data point to BDNF’s potential role as a biological marker or possibly in the pathological mechanisms of burnout. Additionally, these burnout nurse also revealed significant cognitive impairment in reasoning, memory, and attention as well as total scores of CAB. Interestingly, we found a positive correlation between sBDNF levels and the cognitive deficits in burnout nurse, showing that BDNF was positively with reasoning, memory, and attention and CAB total score. These results suggest an interaction between burnout, sBDNF and cognition deficits. However, more research is needed in this regard. The impact on nursing personnel’s cognitive functions has relevant implications and emphasizes the need to implement specific prevention strategies. It is important to consider that nurses make clinical decisions regarding their patients, whose circumstances can alter at any time, and therefore need to constantly rearrange the priorities and tasks of care to accommodate patients’ fluctuating status.

The study has several limitations that should be taken into consideration: 1) Due the sample size among the present study, these results are important to verify in larger samples. 2) This is a cross-sectional design, which hinders establishing a direct causal relationship between BDNF and cognition in burnout nurse. Further long-term research on causal effects is required to clarify the precise nature of the connection between burnout and cognitive function. 3) BDNF levels were measured in serum and despite there are data support the view that BDNF can cross the blood-brain barrier and therefore, the measures of blood and plasma BDNF levels may reflect brain-tissue BDNF levels, the source of peripheral BDNF and its relationships to BDNF in the brain is still unclear. For instance, whole-blood BDNF levels and hippocampus BDNF levels in rats, as well as plasma BDNF and hippocampal BDNF in pigs, were shown to positively correlate, according to Klein et al. [84]. 4) Variables that might impact burnout and cognitive function, like physical exercise, mental health issues, and smoking, were not included in the current study; therefore, these variables should be addressed in future research 5) As previously recognized, exposure to stress hormones (e.g. cortisol) has an impact on BDNF levels and brain structures involved in cognition. However, the study overlooks the evaluation of stress hormones’ impact on BDNF levels and cognition.
